# Downregulation of long noncoding RNA DLEU1 attenuates hypersensitivity in chronic constriction injury-induced neuropathic pain in rats by targeting miR-133a-3p/SRPK1 axis

**DOI:** 10.1186/s10020-020-00235-6

**Published:** 2020-11-10

**Authors:** Zhen Li, Aiyuan Li, Liping Yan, Tian Yang, Wei Xu, Pengju Fan

**Affiliations:** 1 Department of Anesthesiology, Hunan Provincial Maternal and Child Health Care Hospital, Changsha, 410008 Hunan China; 2 Department of Burn and Plastic Surgery, Xiangya Hospital Central South University, No. 87 Xiangya Road, Kaifu District, Changsha, 410008 Hunan China

**Keywords:** DLEU1, miR-133a-3p, SRPK1, Neuropathic pain

## Abstract

**Background:**

Neuropathic pain belongs to chronic pain and is caused by the primary dysfunction of the somatosensory nervous system. Long noncoding RNAs (lncRNAs) have been reported to regulate neuronal functions and play significant roles in neuropathic pain. DLEU1 has been indicated to have close relationship with neuropathic pain. Therefore, our study focused on the significant role of DLEU1 in neuropathic pain rat models.

**Methods:**

We first constructed a chronic constrictive injury (CCI) rat model. Paw withdrawal threshold (PWT) and paw withdrawal latency (PWL) were employed to evaluate hypersensitivity in neuropathic pain. RT-qPCR was performed to analyze the expression of target genes. Enzyme-linked immunosorbent assay (ELISA) was conducted to detect the concentrations of interleukin‐6 (IL-6), tumor necrosis factor‐α (TNF-α) and IL-1β. The underlying mechanisms of DLEU1 were investigated using western blot and luciferase reporter assays.

**Results:**

Our findings showed that DLEU1 was upregulated in CCI rats. DLEU1 knockdown reduced the concentrations of IL‐6, IL‐1β and TNF‐α in CCI rats, suggesting that neuroinflammation was inhibited by DLEU1 knockdown. Besides, knockdown of DLEU1 inhibited neuropathic pain behaviors. Moreover, it was confirmed that DLEU1 bound with miR-133a-3p and negatively regulated its expression. SRPK1 was the downstream target of miR-133a-3p. DLEU1 competitively bound with miR-133a-3p to upregulate SRPK1. Finally, rescue assays revealed that SRPK1 overexpression rescued the suppressive effects of silenced DLEU1 on hypersensitivity in neuropathic pain and inflammation of spinal cord in CCI rats.

**Conclusion:**

DLEU1 regulated inflammation of the spinal cord and mediated hypersensitivity in neuropathic pain in CCI rats by binding with miR-133a-3p to upregulate SRPK1 expression.

## Introduction

Neuropathic pain, a chronic pain caused by nerve lesions or dysfunction, has become the most challenging neurological disease around the world. Neuropathic pain is related to hyperalgesia and dysphonia. More than 20% of cancer pain has close association with neuropathic pain (Bouhassira et al. 2008; Hecke et al. 2014). Neuropathic pain affects many people in the world. However, the molecular regulatory mechanisms in neuropathic pain remain unknown. It is urgent to discover the specific molecular mechanisms and investigate effective therapeutic treatment for neuropathic pain.

Previous studies have reported greater neuropathic pain frequency among females, for example, as indicated by de Mos, the incidence rates for females and males with complex regional pain syndrome were 40.4 and 11.9 per 100,000 person years (Mos 2007). Torrance et al. estimated the prevalence of pain of predominantly neuropathic origin in 6000 adults from 3 United Kingdom cities, and identified that females (6%) showed greater prevalence of neuropathic pain (lasting longer than 3 months) compared with males (3%) (Torrance et al. 2006). Bouhassira et al. used a large sample of the French population to assess neuropathic pain and found higher 3-month prevalence in females (8%) compared with males (6%) (Bouhassira et al. 2008). Consequently, it appears that women are at greater risk for neuropathic pain than men.

Neuroinflammation has been indicated to be closely associated with neuropathic pain (Ellis and Bennett 2013). Activation of glial cells, including satellite glial cells, microglia, and astrocytes in the peripheral and central nervous system contributes to neuroinflammation (Skaper et al. 2017). Microglia, the main immunocompetent cell in the central nervous system that regulates homeostasis in the spinal cord, takes up 5–10% of the glia in the central nervous system (Chen et al. 2018; Yasui 2014). Numerous studies have revealed that activated microglia in the spinal dorsal horn plays a key role in neuropathic pain by releasing pro-inflammatory cytokines including interleukin (IL)-1β, IL-6, tumor necrosis factor (TNF)-α to activate and sensitize spinal cord nociceptive neurons (Leung and Cahill 2010; Kobayashi et al. 2016; Sayo 2019). Thus, the present study used microglia for the in-vitro assays.

Long noncoding RNAs (lncRNAs) are a group of noncoding RNAs with more than 200 nucleotides in length (Mercer et al. 2009). Increasing researches have manifested that lncRNAs serve as core regulators in neuropathic pain. For instance, MALAT1 accelerates neuropathic pain development by targeting the miR‑154‑5p/AQP9 axis in CCI rats (Wu et al. 2019). CRNDE aggravates neuropathic pain development by targeting the miR-136/IL6R axis in CCI rats (Zhang et al. 2019a). Recently, the function of lncRNA deleted in lymphocytic leukemia 1 (DLEU1) has aroused our attention. DLEU1 can upregulate the expression of IGF-1R (Zhang et al. 2019b) and IGF-1R contributes to inflammatory pain sensitivity (Zhang, et al. 2014). DLEU1 can positively regulate ROCK1 (Li et al. 2020a), and the activated ROCK pathway in spinal cord tissues can induce inflammatory pain in mice (Wong et al. 2019). Furthermore, neuropathic pain is a common symptom of multiple sclerosis (Yousuf, et al. 2020), and rs9596270 of DLEU1 is identified as susceptibility risk factor for Greek multiple sclerosis (Hadjigeorgiou 2019). In this study, we focused on the role of DLEU1 and its molecular regulatory mechanism in neuropathic pain progression, which may offer a more extensive method for the treatment of neuropathic pain.

## Materials and methods

### Animal studies

Compared with men, women are more vulnerable to pain. To yield results that might work for women, female rats were used in the present study. Adult female Sprague–Dawley rats weighed 180–200 g were obtained from Vital River company (Beijing, China). All experiments were conducted in line with the requirements of national institutes of health guide for the care and use of laboratory animals. The rats were divided into six groups, with eight rats in each group. The rats were raised in a constant feeding environment at 20–25 ℃. One week later, the rats were categorized into sham operation group and chronic constriction injury (CCI) group. The intrathecal administration was conducted before CCI surgery for gene delivery. Finally, the spinal cord horn tissues (L4–L5) were collected for next experiments after rats were sacrificed.

### Intrathecal injection

Intrathecal injection was performed according to a previous study (Cheng et al. 2020). Briefly, the rat occipital muscles were isolated. Then the PE-10 polyethylene catheter was inserted in the cisterna magna of the rats. The incision was sealed after the catheter was fixed. To examine whether the catheter was inserted successfully, 20 μL of lidocaine was intrathecally injected over 30 s followed by a 10-μL flush of normal saline (Zhang et al. 2020; Qiu 2020). Hind paw paralysis within 30 s and lasting within 10 min indicated a successful catheterization. Two days later, the rats with movement and sensory disturbances were excluded. For gene delivery, 10 μL of lentivirus (Genepharma, Shanghai, China) containing sh-DLEU1, sh-NC, SRPK1, respectively, was injected into rats via intrathecal catheter with a microinjection syringe 3 days before surgery.

### Cell culture

Rat microglial cells were purchased from Sciencell Research Laboratories and HEK-293T cells were obtained from the American Type Culture Collection (Manassas, USA). Cells were cultured in Dulbecco's modified Eagle medium (DMEM; Lonza Inc. USA) with 10% heat-inactivated fetal bovine serum (FBS; Invitrogen, USA) and 1% penicillin/streptomycin (Sigma, St. Louis, USA). Cells were incubated in the humid atmosphere with 5% CO_2_ at 37 ℃. Spinal cord astrocytes were isolated and cultured according to a previous study (Kerstetter and Miller 2012). Spinal sensory neurons were isolated and cultured as revealed in a previous study (Olschewski et al. 2001).

### Cell transfection

For the downregulation of DLEU1, short hairpin RNAs targeting DLEU1 (sh-DLEU1) or short hairpin negative control (sh-NC) (RiboBio, Guangzhou, China) were transfected into rat microglial cells using Lipofectamine 2000 reagent (Invitrogen, USA) under the instructions of manufacturer. MiR-133a-3p mimics, miR-133a-3p inhibitor or their negative controls (NC mimics and NC inhibitor) were transfected into rat microglial cells.

### Neuropathic pain animal model

Chronic constriction injury (CCI) method was conducted to construct the animal model of neuropathic pain (Miao et al. 2020). The rats were anesthetized with intraperitoneal injection of sodium pentobarbital (40 mg/kg). A mid-thigh incision was conducted to expose the sciatic nerve on the two sides. Then, 4 ligatures using 4-0 chromic gut sutures at the interval of 1 mm were loosely tied around the sciatic nerve around to the sciatic notch until a brief twitch was elicited in the hind limb. The rats with sciatic nerve exposures without ligation served as control groups. The dorsal spinal cords of the rats were collected at Day 0, 7, 14, and 21.

### Pain threshold assessment

Paw withdrawal threshold (PWT) and paw withdrawal latency (PWL) were employed to evaluate hypersensitivity in neuropathic pain (Li 2017). The rats were kept in a transparent plastic box with a metal mesh floor. The plantar surface of each hind paw was exposed to pressure, which was made by calibrated Electronicvon Frey filament (Electronic von Frey 2393; IITC, Woodland Hills, USA). Next, the force upon paw withdrawal was recorded. PWL was utilized to evaluate thermal hypersensitivity responding to radiant heat. The heat intensity was set at 45 ℃ and the cut-off time was at 30 s. Then the duration between stimuli starting and paw withdrawal was evaluated.

### Real-time quantitative polymerase chain reaction (RT-qPCR)

Total RNA was extracted utilizing the TRIzol reagent (Takara, Dalian, China) in line with the manufacturer’s instructions. The RNA was reversely transcribed to cDNA utilizing a First Strand cDNA Synthesis Kit (Fermentas, Canada). The RT-qPCR was performed via a SYBR premix Ex Taq kit (TaKaRa, Dalian, China). The results of RT-qPCR were evaluated utilizing Thermal Cycler Dice Real Time PCR System (Takara, Dalian, China). The 2^−∆∆Ct^ method was utilized to calculate the relative expression of target genes (Zheng 2016). Internal controls were GAPDH and U6. The sequences of primers were as follows:

DLEU1: 5′‐TGCATTTAAAACCGCCCTGC‐3′ (forward),

DLEU1: 5′‐TTGAAGAAGGAGACCACGCC‐3′ (reverse);

miR-133a-3p: 5′‐GGGAGCCAAATGCTTTGCTAG‐3′ (forward),

miR-133a-3p:5′‐CCCTCGGTTTACGAAACGATC‐3′ (reverse);

SRPK1: 5′‐CCACGCTCTTCGCCATTC‐3′ (forward),

SRPK1: 5′‐GAGACCGGTAACCGCCAG‐3′ (reverse);

GAPDH: 5′‐CAAGGTCATCCATGACAACTTTG‐3′ (forward),

GAPDH: 5′‐GTCCACCACCCTGTTGCTGTAG‐3′ (reverse);

U6: 5′‐CTCGCTTCGGCAGCACA‐3′ (forward),

U6: 5′‐AACGCTTCACGAATTTGCGT‐3′ (reverse).

### Western blot analysis

The total protein was extracted utilizing Protein Lysis Buffer. The protein quality was evaluated by BCA method. The protein was run on 8% sodium dodecyl sulfate‐polyacrylamide gels (SDS-PAGE). Then separated protein was transferred to the polyvinylidene difluoride (PVDF) membrane. The membrane was blocked in 5% defatted milk for 1 h. Next, the primary antibodies (Abcam, Cambridge, UK) were added into membranes, and the membranes were incubated overnight at 4 ℃. After three times washing by Tris-buffered saline Tween-20, the membranes were cultured with the secondary antibodies (ab205718; 1:2000) for 1 h. Protein was detected via an enhanced chemiluminescence detection kits (BB-3501, Amersham Pharmacia Biotech, UK) and Bio-Rad image analysis system (Bio-Rad Laboratories, Inc. USA). The primary antibodies were anti-SRPK1 (ab189839; 1:500), anti-GAPDH (ab181602; 1:10,000).

### Luciferase activity assay

The wild-type (Wt) or mutant (Mut) DLEU1 binding site of miR-133a-3p was designed and subcloned into pmirGLO Basic vector (Promega, USA) to construct the pmirGLO-DLEU1-Wt or pmirGLO-DLEU1-Mut plasmid. Similarly, Wt or Mut 3′-UTR of SRPK1 was subcloned into pmirGLO Basic vector to construct the pmirGLO-SRPK1-Wt or pmirGLO-SRPK1-Mut plasmid. HEK-293T cells were cultured on 24-well plates. Then, miR-133a-3p mimics were co-transfected with pmirGLO-DLEU1-Wt or pmirGLO-DLEU1-Mut, pmirGLO-SRPK1-Wt or pmirGLO-SRPK1-Mut plasmids into HEK-293T cells by Lipofectamine 2000. 48 h later, the relative luciferase activities were determined by luciferase reporter assay system (Promega, USA).

### Enzyme-linked immunosorbent assay (ELISA)

The concentrations of IL-6, TNF-α and IL-1β were detected by ELISA kits (R&D Systems, USA) according to the manufacturer's protocols.

### RNA-fluorescence in situ hybridization (FISH) assay

The subcellular localization of DLEU1 in cells was detected via a FISH assay. DLEU1 probes were designed and synthesized by RiboBio (Guangzhou, China). The probe signals were detected with a FISH Kit (RiboBio, Guangzhou, China) based on the manufacturer’s instructions. In brief, the slides were separated and washed with PBS. Then the slides were fixed with 4% paraformaldehyde. Next the cells were treated with protease K, glycine and acetylation reagent, followed by adding with prehybridization solution and cultured for 1 h. Then the cells were hybridized with lncRNA DLEU1 (300 ng/mL) hybrid solution containing probe overnight at 37 ℃. Subsequently, the nucleus was stained with DAPI solution (ab104139, 1:100, Abcam, Shanghai, China). Finally, an anti-fluorescent quenching agent was added into the cells, and images were detected by a fluorescence microscope.

### Statistical analysis

Statistical analysis was conducted with GraphPad Prism 6.0 software. Data are exhibited as the mean ± SD. Student's *t* test was used to analyze data between 2 groups, while the comparison among multiple groups was evaluated by one‐way analysis of variance. All experiments were repeated three times. *p* < 0.05 was considered significant.

## Results

### DLEU1 was upregulated in CCI rats

**Fig. 1:**
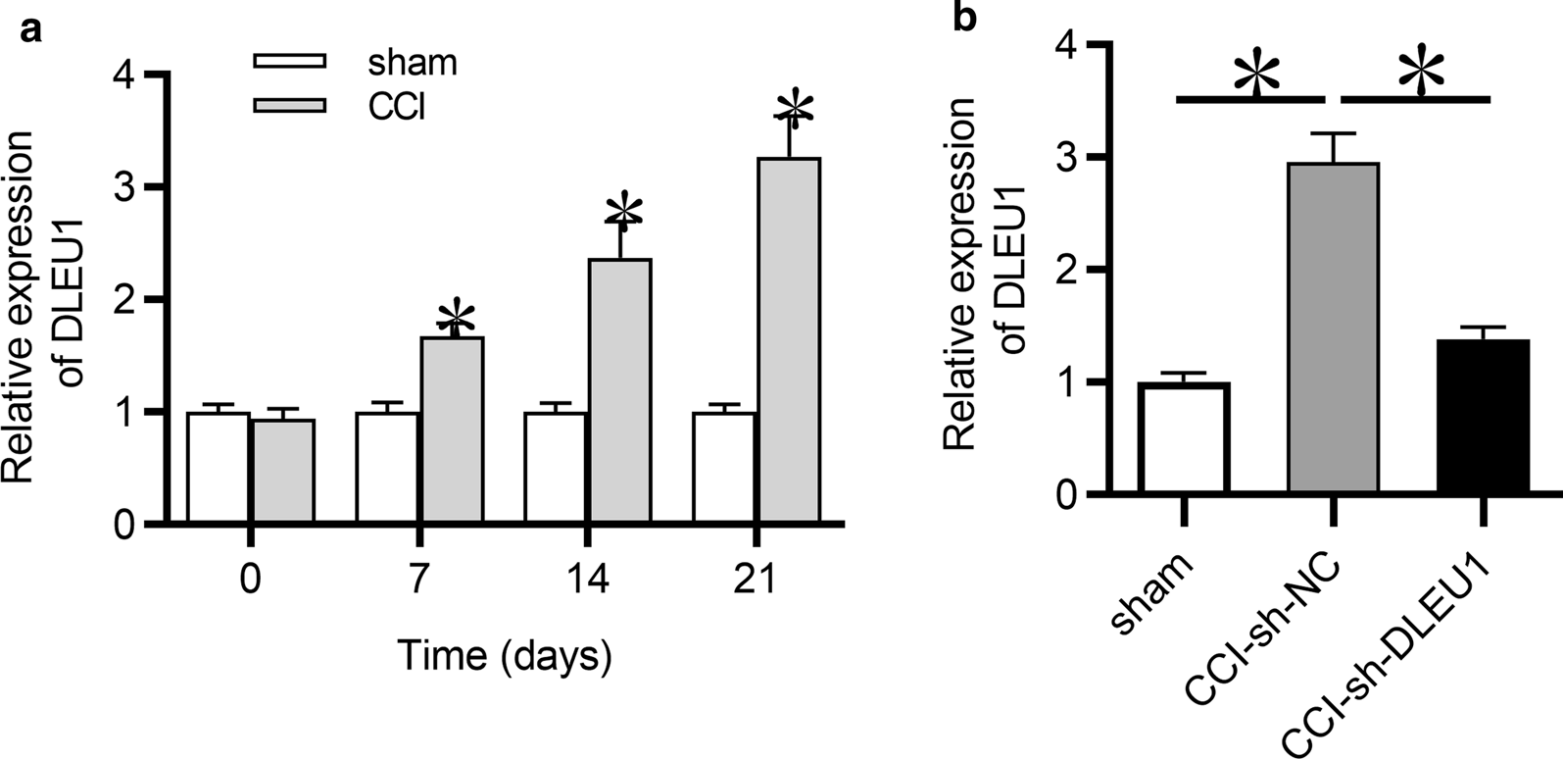
DLEU1 was upregulated in CCI rats. **a** DLEU1 expression in the dorsal spinal cord of rats was detected by RT-qPCR analysis at postoperative days 0, 7, 14 and 21. N = 8 for each group. **b** Expression of DLEU1 in CCI rat models infected with lentivirus carrying sh-DLEU1 or sh-NC at postoperative day 7 was detected by RT-qPCR analysis. N = 8 for each group. *p < 0.05

First, DLEU1 expression in the spinal cord tissues of rats was detected by RT-qPCR. As shown in Fig. 1a, DLEU1 expression was significantly higher in CCI rats compared to the sham group at postoperative days 7, 14 and 21, suggesting that DLEU1 might participate in CCI induced neuropathic pain. Next, DLEU1 was knocked down in CCI rats by intrathecal injection of lentivirus carrying sh-DLEU1. RT-qPCR analysis indicated that DLEU1 expression was effectively knocked down in spinal cord tissues of CCI rats, compared to that in CCI-sh-NC group (Fig. 1b).

### DLEU1 knockdown suppressed inflammation in spinal cord tissues and attenuated neuropathic pain-like behaviors

**Fig. 2:**
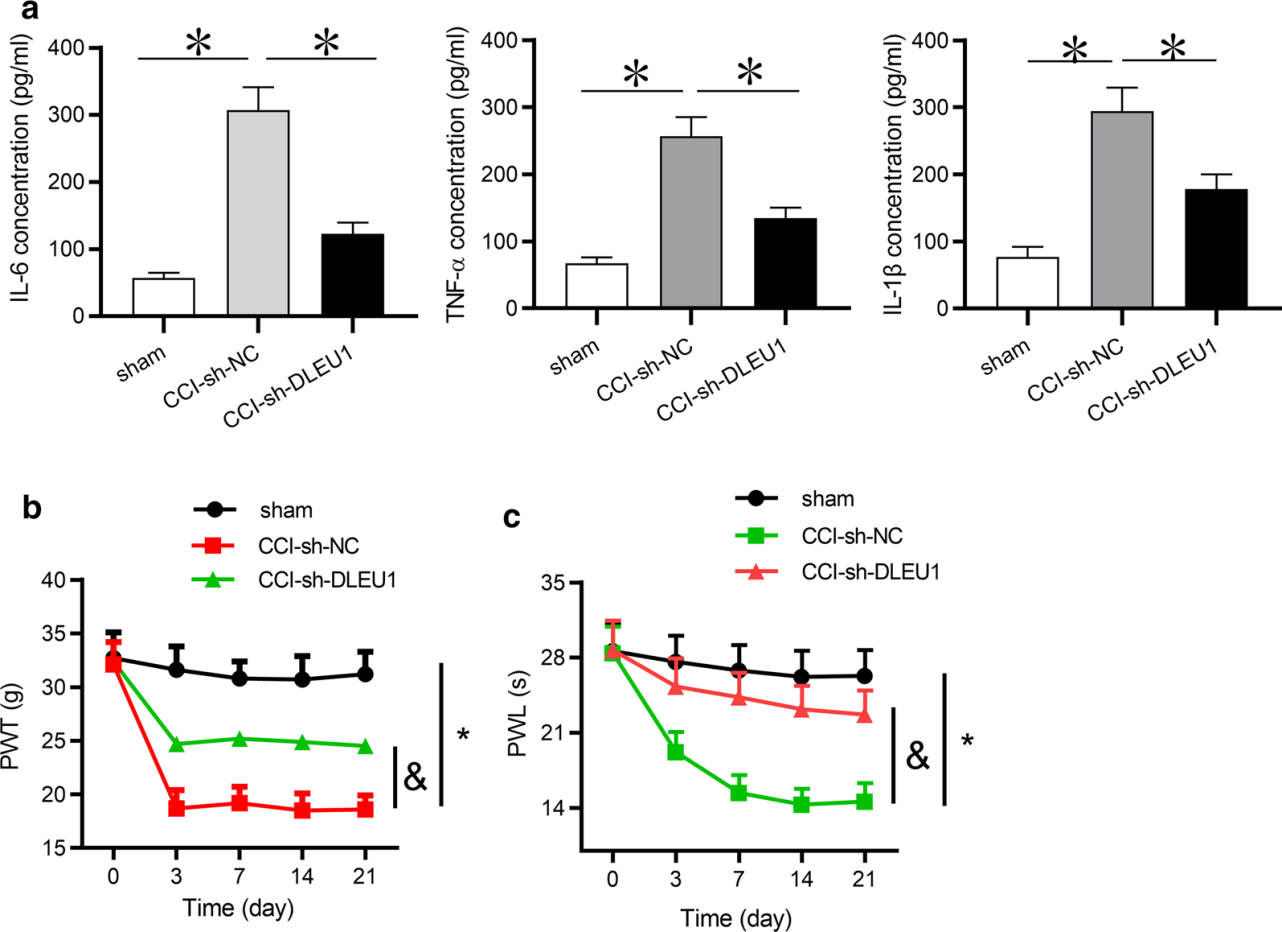
DLEU1 knockdown suppressed neuroinflammation and attenuated neuropathic pain-like behaviors. **a** The concentrations of IL‐6, TNF‐α and IL-1β in the dorsal spinal cord of rats were detected by ELISA at postoperative Day 7. N = 8 for each group. **b** The effect of DLEU1 knockdown on behavioral nociceptive responses to mechanical stimuli was assessed by paw withdrawal mechanical threshold (PWT) at postoperative days 0, 7, 14 and 21. N = 8 for each group. **c** The effect of DLEU1 knockdown on behavioral nociceptive responses to thermal hypersensitivity was evaluated by paw withdrawal latency (PWL) at postoperative days 0, 7, 14 and 21. N = 8 for each group. *p < 0.05, ^&^p < 0.05

Since IL-6, TNF-α and IL-1β are inflammation-associated cytokines and exert important function in neuropathic pain, the concentrations of inflammatory cytokines including IL-6, TNF-α and IL-1β were detected in spinal cord tissues of CCI rats after DLEU1 was knocked down. Results from ELISA showed that the concentrations of IL-6, IL-1β and TNF-α were reduced by the inhibition of DLEU1 in CCI rats (Fig. 2a), suggesting that DLEU1 knockdown inhibited neuroinflammation in CCI rats. Moreover, neuropathic pain-like behaviors were reduced by the silencing of DLEU1 in CCI rat models (Fig. 2b, c). Furthermore, we explored the effects of DLEU1 alone on neuropathic pain-like behaviors in rats receiving no CCI surgery. The results depicted that DLEU1 alone had no significant influence on neuropathic pain-like behaviors in sham rats (Additional file 1: Fig. S1A, B).

### DLEU1 bound with miR-133a-3p

**Fig. 3:**
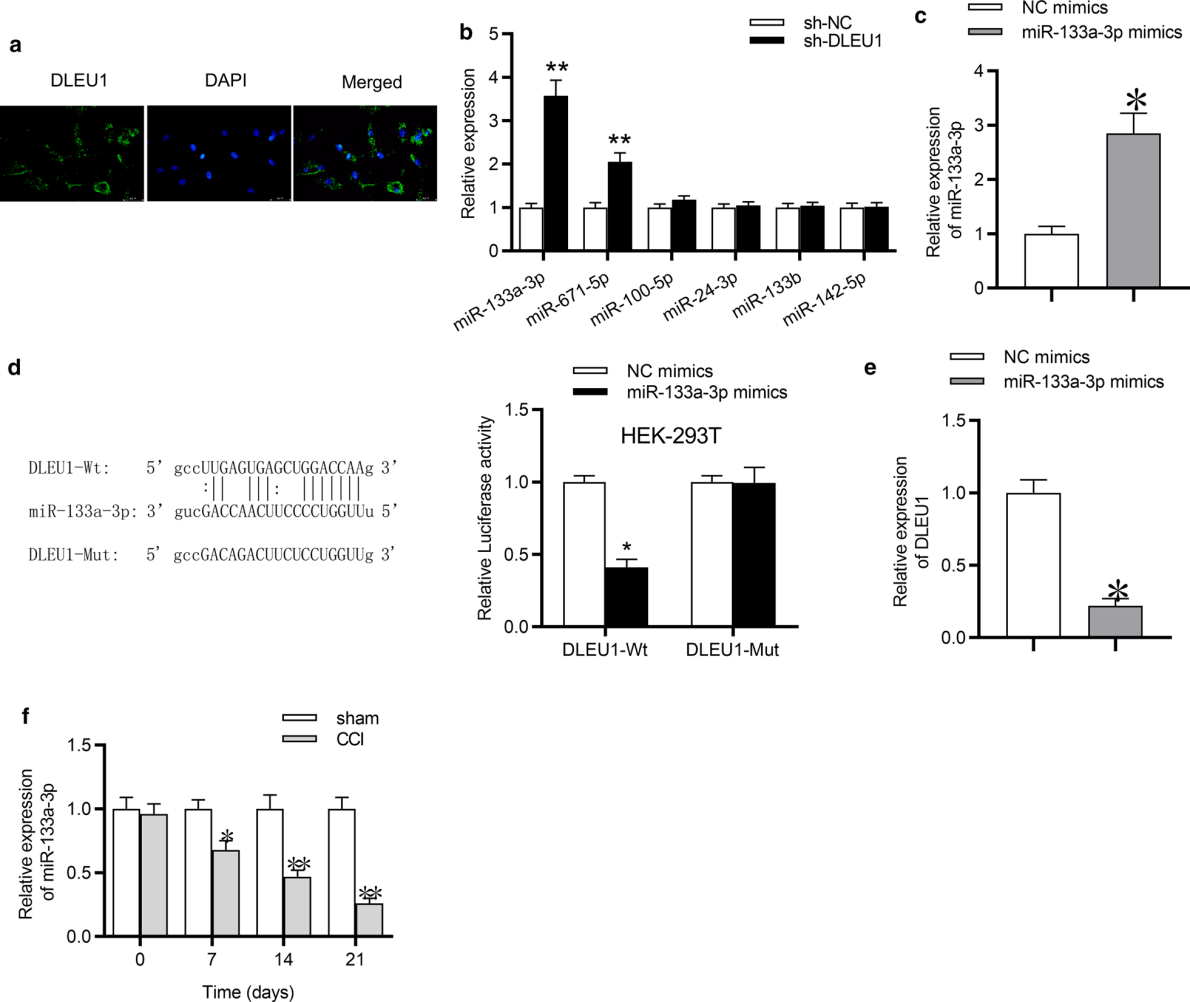
DLEU1 bound with miR-133a-3p. **a** FISH assay was conducted to detect the DLEU1 subcellular localization in rat microglial cells. **b** RT-qPCR analysis was conducted to detect the relative expression of miRNAs in rat microglial cells by indicated transfections. **c** MiR-133a-3p expression in rat microglial cells was overexpressed by transfection with miR-133a-3p mimics and the efficiency of transfection was determined by RT-qPCR analysis. **d** Luciferase reporter assay was conducted to confirm the relationship between miR-133a-3p and DLEU1 in HEK-293T cells. **e** RT-qPCR analysis was conducted to detect the expression of DLEU1 in rat microglial cells transfected with miR-133a-3p mimics. **f** RT-qPCR analysis was conducted to assess the expression of miR-133a-3p in spinal cord tissues after CCI surgery for 0, 7, 14, 21 days. *p < 0.05, **p < 0.01

Since DLEU1 was reported to serve as a competitive endogenous RNA (ceRNA) at the post-transcriptional level in various diseases (Chen et al. 2019; Feng et al. 2019; Li et al. 2020b), we aimed to explore whether DLEU1 can serve as a ceRNA in neuropathic pain. Initially, RNA FISH was utilized to detect the subcellular localization of DLEU1 in rat microglial cells, and the results manifested that DLEU1 was primarily located in the cytoplasm of microglial cells (Fig. 3a), suggesting the potential ceRNA role of DLEU1. Subsequently, we searched the starBase online websites for the potential target of DLEU1, and six miRNAs were selected (screening condition: Pan-Cancer: 8 cancer types). Next, RT-qPCR analysis implied that DLEU1 knockdown triggered the most significant upregulation of miR-133a-3p expression in rat microglial cells and in spinal cord tissues of CCI rats compared with other miRNAs (Fig. 3b; Additional file 1: Fig. S1C), thus miR-133a-3p was selected from the candidate miRNAs for further investigation. In addition, miR-133a-3p expression was overexpressed by transfection of miR-133a-3p mimics in rat microglial cells (Fig. 3c). Moreover, the binding sites between DLEU1 and miR-133a-3p were predicted by starBase. Next, luciferase reporter assay suggested that the luciferase activity of pmirGLO-DLEU1-Wt plasmids was reduced by miR-133a-3p overexpression, while there was no significant change on the luciferase activity of pmirGLO-DLEU1-Mut plasmids in HEK‐293T cells (Fig. 3d). From Fig. 3e, RT-qPCR analysis indicated that miR-133a-3p overexpression suppressed the expression of DLEU1 in rat microglial cells. Furthermore, the expression of miR-133a-3p was downregulated in spinal cord tissues of CCI rats compared with sham group at postoperative days 7, 14, and 21 (Fig. 3f).

### SRPK1 was the downstream target of miR-133a-3p

**Fig. 4:**
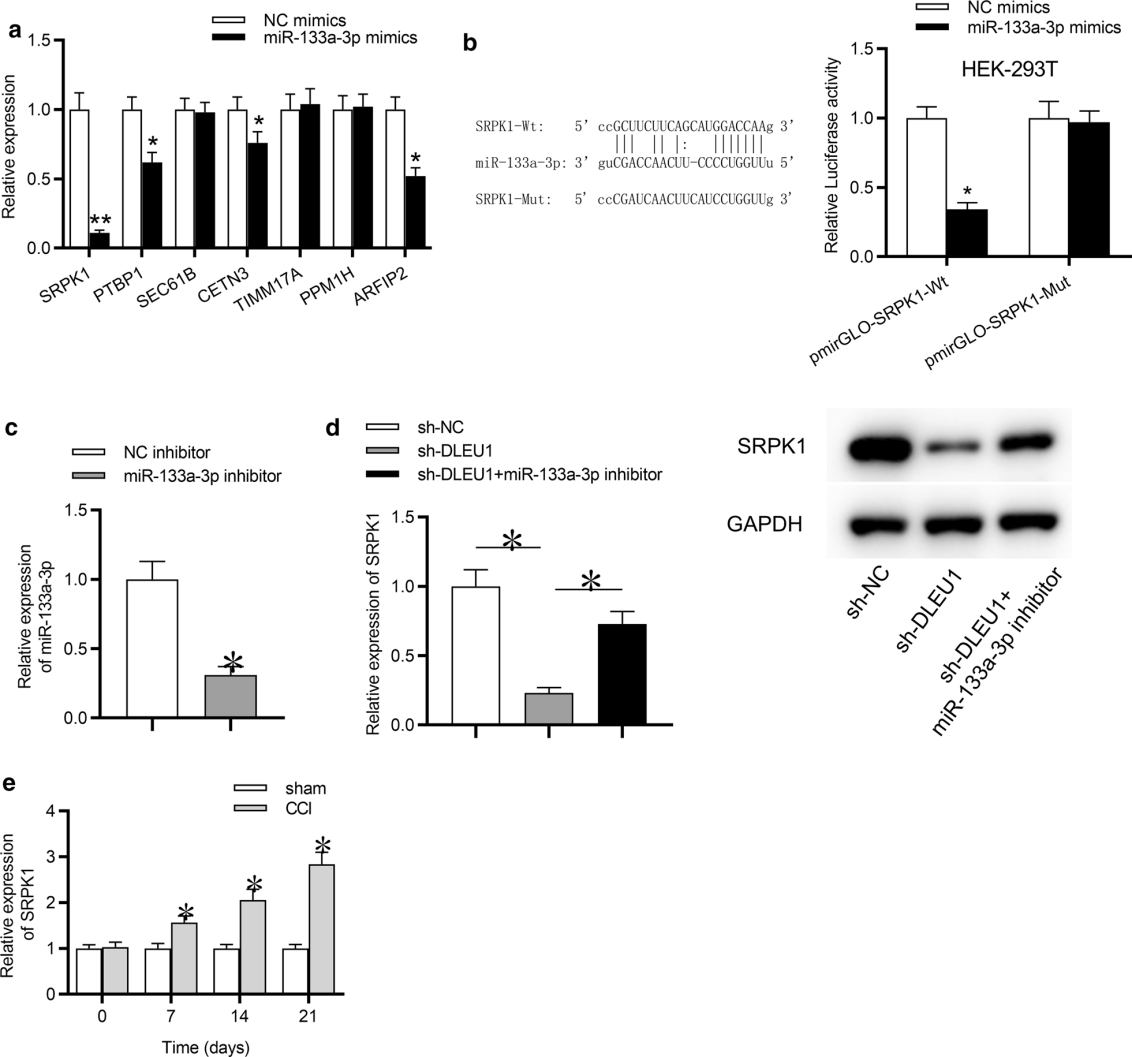
SRPK1 was the downstream target of miR-133a-3p. **a** RT-qPCR analysis was conducted to detect the expression of target genes regulated by miR-216a-5p upregulation in rat microglial cells. **b** Luciferase reporter assay was conducted to explore the relationship between miR-133a-3p and SRPK1 in HEK-293T cells. **c** Expression of miR-133a-3p in rat microglial cells transfected with miR-133a-3p inhibitor or NC inhibitor was detected by RT-qPCR analysis. **d** RT-qPCR and western blot analyses were conducted to detect the mRNA or protein expression of SRPK1 in rat microglial cells by indicated transfections. **e** RT-qPCR analysis was conducted to assess the expression of SRPK1 in spinal cord tissues after CCI surgery for 0, 7, 14, 21 days. *p < 0.05

MiRNAs were reported to exert effects on neuropathic pain by targeting specific genes (Ji et al. 2018). Hence, we sought to find out the downstream target of miR-133a-3p. We searched the TargetScan online website and found seven mRNAs that can bind with miR-133a-3p. Next, RT-qPCR analysis implied that miR-133a-3p overexpression triggered the most significant downregulation of SRPK1 in rat microglial cells, while expression of other 6 mRNAs was not impacted in response to miR-133a-3p overexpression (Fig. 4a). Thus, SRPK1 was selected from the candidate mRNAs for further investigation. Subsequently, the binding sites between miR-133a-3p and SRPK1 were predicted by TargetScan. The following luciferase reporter assay suggested that the luciferase activity of pmirGLO-SRPK1-Wt plasmid was reduced by miR-133a-3p overexpression, while there was no significant change on the luciferase activity of pmirGLO-SRPK1-Mut plasmid in HEK‐293T cells (Fig. 4b). From Fig. 4c, RT-qPCR analysis indicated that miR-133a-3p expression was effectively knocked down by miR-133a-3p inhibitor in rat microglial cells. The mRNA and protein expression levels of SRPK1 were inhibited by DLEU1 depletion in rat microglial cells, while these effects were reversed by the introduction of miR-133a-3p inhibitor (Fig. 4d). Furthermore, the expression of SRPK1 was upregulated in spinal cord tissues of CCI rats compared with sham group at postoperative days 7, 14, and 21 (Fig. 4e). Moreover, we compared the expression of DLEU1, miR-133a-3p and SRPK1 in spinal cord dorsal horn ipsilateral to the CCI with that in the contralateral segments. As revealed in Additional file 1: Fig. S1D, DLEU1 and SRPK1 were significantly upregulated in ipsilateral segments compared to that in contralateral segments, while miR-133a-3p was significantly downregulated in ipsilateral segments compared to that in contralateral segments. Moreover, astrocytes and neuronal cells were isolated from the CCI rats. Expression of DLEU1 and SRPK1 in astrocytes and neuronal cells was compared to that in microglia. As revealed in Additional file 1: Fig. S1E, F, DLEU1 and SRPK1 expression displayed no significant difference in astrocytes, compared to that in microglia. DLEU1 and SRPK1 were significantly underexpressed in neuronal cells, compared to that in microglia.

### Overexpression of SRPK1 reversed the effects of silenced DLEU1 on inflammation and neuropathic pain-like behaviors in CCI rats

**Fig. 5:**
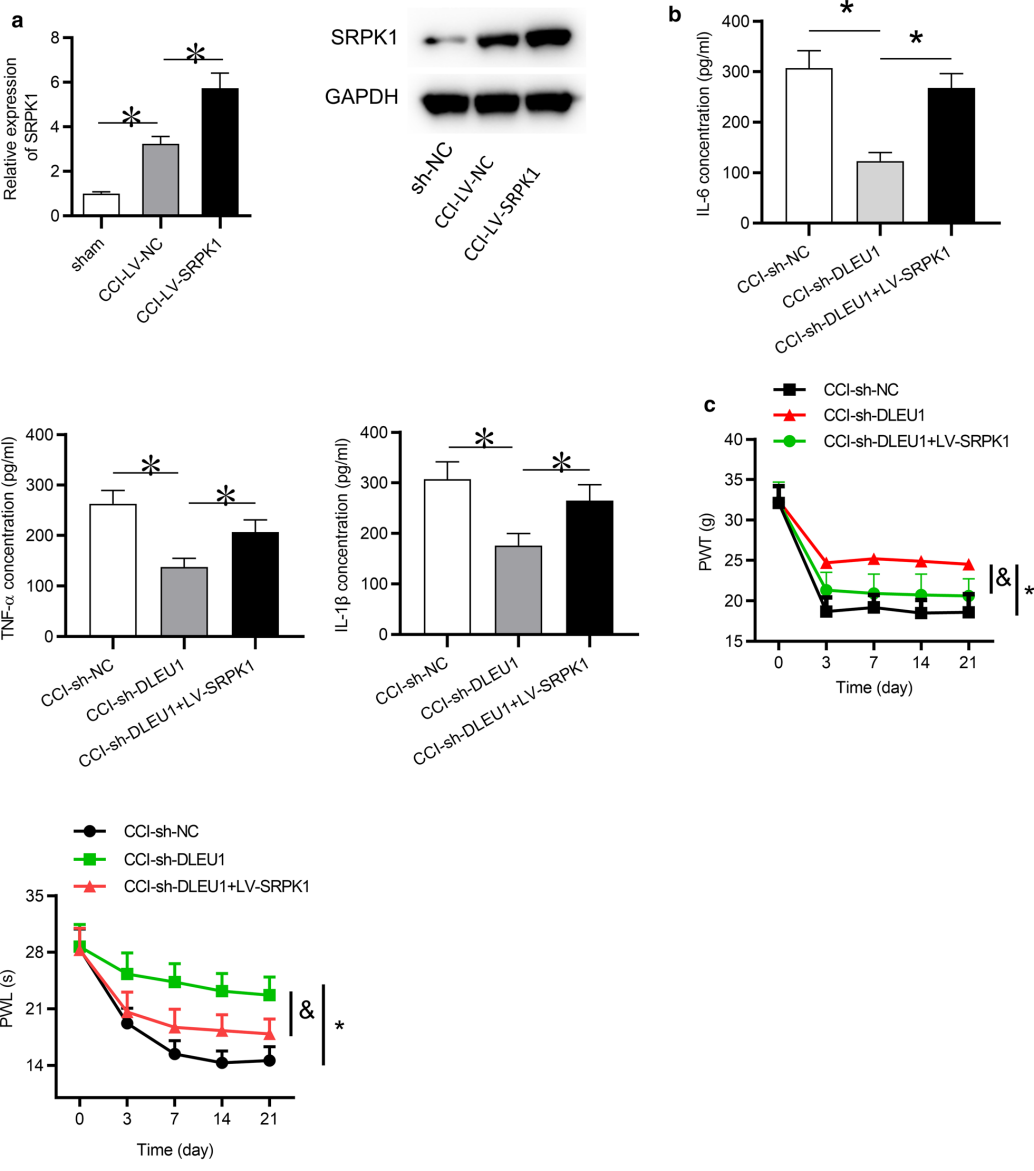
Overexpression of SRPK1 reversed the effects of silenced DLEU1 on inflammation and neuropathic pain-like behaviors in CCI rats. **a** The mRNA or protein expression of SRPK1 in CCI rats spinal cord tissues infected with lentivirus encoding SRPK1 at postoperative day 7 was detected by RT-qPCR analysis. N = 8 for each group. **b** ELISA tested the concentrations of IL‐6, TNF-α and IL‐1β in spinal cord tissues of CCI rats at postoperative day 7. N = 8 for each group. **c** Paw withdrawal mechanical threshold (PWT) at postoperative days 0, 7, 14 and 21 days. N = 8 for each group. **d** The thermal hypersensitivity was assessed by paw withdrawal latency (PWL) at postoperative days 0, 7, 14 and 21 days. N = 8 for each group. *p < 0.05, ^&^p < 0.05

Finally, whether DLEU1 regulates inflammation and neuropathic pain-like behaviors in CCI rats by upregulation of SRPK1 was investigated. SRPK1 was first overexpressed in spinal cord tissues of CCI rats by intrathecal injection of lentivirus encoding SRPK1. RT-qPCR and western blot analyses showed that the mRNA and protein expression levels of SRPK1 were upregulated by delivery of LV-SPRK1 (Fig. 5a). Subsequently, ELISA showed that SRPK1 overexpression countervailed the inhibitive influence of DLEU1 knockdown on the concentrations of IL-6, TNF-α and IL-1β in CCI rats (Fig. 5b). Moreover, the attenuating effects of silenced DLEU1 on neuropathic pain-like behaviors in CCI rats were counteracted by SRPK1 overexpression (Fig. 5c, d).

## Discussion

Neuropathic pain is related to local neuroinflammation in the spinal cord, which may lead to disability and induce a severe socioeconomic burden (Dominguez et al. 2010). Cytokines act as significant modulators of inflammatory response and participate in the pathophysiological processes of neuropathic pain (Ellis and Bennett 2013). IL-6, IL-1β and TNF-α have been reported to act as pro-inflammatory cytokines and play vital roles in neuropathic pain (Li et al. 2017). Previous studies have demonstrated that lncRNAs serve as core regulators in neuropathic pain (Wu et al. 2019; Zhang et al. 2019a). In the present study, a CCI rat model was established and we found that DLEU1 was upregulated in spinal cord tissues of CCI rats. The concentrations of IL‐6, TNF‐α, IL-1β were inhibited by DLEU1 knockdown. Moreover, neuropathic pain-like behaviors were reduced by the silencing of DLEU1 in CCI rat models.

MicroRNAs (miRNAs) are small non-coding RNAs at a length of about 21–25 nucleotide (Bartel 2004). Recently, miRNAs have been reported to be abnormally expressed in the nervous system and exert essential effects to modulate neuropathic pain. For example, miR-183 overexpression inhibits neuropathic pain by targeting MAP3K4 in CCI rat models (Huang and Wang 2019). MiR-101 alleviates neuropathic pain in CCI rat models by downregulating mTOR expression (Xie et al. 2019). Furthermore, lncRNAs have been reported to bind with specific miRNAs and modulate neuropathic pain. For example, XIST aggravates neuropathic pain development by modulating miR-150 and ZEB1 in CCI rat models (Yan 2018). NEAT1 accelerates neuropathic pain progression by targeting the miR-381/HMGB1 axis in CCI rat models (Xia et al. 2018). In our study, it was confirmed that DLEU1 bound with miR-133a-3p, and miR-133a-3p expression was downregulated in spinal cord tissues of CCI rats. MiR-133a-3p is differentially expressed 2 weeks after induced Müller cell ablation, which can cause neuroinflammation (Chung 2015). MiR-133a-3p is significantly downregulated in the M1 or M2a-skewed microglia (Freilich et al. 2013). Neuropathic pain is a common complication of diabetes, and patients with type-2 diabetes mellitus express decreased levels of miR-133a-3p (Kokkinopoulou 2019).

Serine/arginine protein kinase 1 (SRPK1) is a multifunctional protein, and participate in various cellular activities, such as cell cycle development, innate immune response, and inflammation (Tunnicliffe et al. 2019). Thus, increasing researchers focused on exploring the biological role of SRPK1 in human diseases. Inhibition of SRPK1 in the spinal cord attenuated behavioral nociceptive responses to mechanical and heat stimuli (Hulse et al. 2016). Pharmacological inhibition of SRPK1 after traumatic nerve injury reduces VEGF-Axxxa expression and reversed associated neuropathic pain (Hulse 2014). In the present study, SRPK1 was confirmed to be the downstream target of miR-133a-3p, and SRPK1 was upregulated in CCI rats. DLEU1 upregulated expression of SRPK1 via binding with miR-133a-3p. Finally, rescue assays revealed that SRPK1 countervailed DLEU1 mediated regulation on inflammation and neuropathic pain-like behaviors in CCI rats.

## Conclusion

The present study illustrated that DLEU1 bound with miR-133a-3p to antagonize the inhibitory effects of miR-133a-3p on SRPK1, and thus upregulated SRPK1 expression. Downregulation of DLEU1 reduced inflammation of the spinal cord and alleviated hypersensitivity in neuropathic pain in CCI rats, which may provide evidence for therapy protocols of neuropathic pain.

## Supplementary information

**Additional file 1: Fig. S1.** (A-B) PWT and PWL were evaluated in DLEU1 silenced sham rats (compared to sham rats without any other treatments) on Day 3, 6, 9, 12 post delivery of LV-sh-DLEU1. N=8 for each group. (C) Relative expression of 6 candidate miRNAs in spinal cord tissues of rats at postoperative day 7 of CCI was examined by RT-qPCR analysis. N=8 for each group. (D) Relative expression of DLEU1, miR-133a-3p and SRPK1 in spinal cord dorsal horn contralateral to the CCI surgery (compared to ipsilateral segments) was examined by RT-qPCR analysis. N=8 for each group. (E-F) Relative expression of DLEU1 and SRPK1 in astrocytes and neuronal cells (compared to microglia) isolated from CCI rats was detected by RT-qPCR analysis. *p < 0.05.

## Data Availability

The datasets used and analyzed during the current study are available from the corresponding author on reasonable request.

## References

[CR1] Bartel DP (2004). MicroRNAs: genomics, biogenesis, mechanism, and function. Cell.

[CR2] Bouhassira D, Lantéri-Minet M, Attal N, Laurent B, Touboul C (2008). Prevalence of chronic pain with neuropathic characteristics in the general population. Pain.

[CR3] Chen G, Zhang YQ, Qadri YJ, Serhan CN, Ji RR (2018). Microglia in pain: detrimental and protective roles in pathogenesis and resolution of pain. Neuron.

[CR4] Chen X, Zhang C, Wang X (2019). Long noncoding RNA DLEU1 aggravates osteosarcoma carcinogenesis via regulating the miR-671-5p/DDX5 axis. Artif Cells Nanomed Biotechnol.

[CR5] Cheng H (2020). Astrocytic NDRG2 is critical in the maintenance of neuropathic pain. Brain Behav Immun.

[CR6] Chung SH (2015). Profiling of microRNAs involved in retinal degeneration caused by selective Müller cell ablation. PLoS ONE.

[CR7] de Mos M (2007). The incidence of complex regional pain syndrome: a population-based study. Pain.

[CR8] Dominguez E, Mauborgne A, Mallet J, Desclaux M, Pohl M (2010). SOCS3-mediated blockade of JAK/STAT3 signaling pathway reveals its major contribution to spinal cord neuroinflammation and mechanical allodynia after peripheral nerve injury. J Neurosci.

[CR9] Ellis A, Bennett DL (2013). Neuroinflammation and the generation of neuropathic pain. Br J Anaesth.

[CR10] Feng L, He M, Rao M, Diao J, Zhu Y (2019). Long noncoding RNA DLEU1 aggravates glioma progression via the miR-421/MEF2D axis. OncoTargets Ther.

[CR11] Freilich RW, Woodbury ME, Ikezu T (2013). Integrated expression profiles of mRNA and miRNA in polarized primary murine microglia. PLoS ONE.

[CR12] Hadjigeorgiou GM (2019). Replication study of GWAS risk loci in Greek multiple sclerosis patients. Neurol Sci.

[CR13] Huang L, Wang L (2019). Upregulation of miR-183 represses neuropathic pain through inhibiton of MAP3K4 in CCI rat models. J Cell Physiol.

[CR14] Hulse RP (2014). Regulation of alternative VEGF-A mRNA splicing is a therapeutic target for analgesia. Neurobiol Dis.

[CR15] Hulse RP, Drake RA, Bates DO, Donaldson LF (2016). The control of alternative splicing by SRSF1 in myelinated afferents contributes to the development of neuropathic pain. Neurobiol Dis.

[CR16] Ji LJ, Shi J, Lu JM, Huang QM (2018). MiR-150 alleviates neuropathic pain via inhibiting toll-like receptor 5. J Cell Biochem.

[CR17] Kerstetter AE, Miller RH (2012). Isolation and culture of spinal cord astrocytes. Methods Mol Biol (Clifton, N.J.).

[CR18] Kobayashi M, Konishi H, Sayo A, Takai T, Kiyama H (2016). TREM2/DAP12 signal elicits proinflammatory response in microglia and exacerbates neuropathic pain. J Neurosci.

[CR19] Kokkinopoulou I (2019). Decreased expression of microRNAs targeting type-2 diabetes susceptibility genes in peripheral blood of patients and predisposed individuals. Endocrine.

[CR20] Leung L, Cahill CM (2010). TNF-alpha and neuropathic pain–a review. J Neuroinflamm.

[CR21] Li J (2017). Urotensin II inhibitor eases neuropathic pain by suppressing the JNK/NF-κB pathway. J Endocrinol.

[CR22] Li QY, Xu HY, Yang HJ (2017). Effect of proinflammatory factors TNF-alpha, IL-1beta, IL-6 on neuropathic pain. Zhongguo Zhong yao za zhi = Zhongguo zhongyao zazhi = China journal of Chinese materia medica.

[CR23] Li R, Wan T, Qu J, Yu Y, Zheng R (2020). Long non-coding RNA DLEUI promotes papillary thyroid carcinoma progression by sponging miR-421 and increasing ROCK1 expression. Aging.

[CR24] Li H, Huang J, Yu S, Lou Z (2020). Long Non-coding RNA DLEU1 up-regulates BIRC6 expression by competitively sponging miR-381-3p to promote cisplatin resistance in nasopharyngeal carcinoma. OncoTargets Ther.

[CR25] Mercer TR, Dinger ME, Mattick JS (2009). Long non-coding RNAs: insights into functions. Nat Rev Genet.

[CR26] Miao J, Zhou X, Ji T, Chen G (2020). NF-κB p65-dependent transcriptional regulation of histone deacetylase 2 contributes to the chronic constriction injury-induced neuropathic pain via the microRNA-183/TXNIP/NLRP3 axis. J Neuroinflamm.

[CR27] Olschewski A, Hempelmann G, Vogel W, Safronov BV (2001). Suppression of potassium conductance by droperidol has influence on excitability of spinal sensory neurons. Anesthesiology.

[CR28] Qiu S (2020). MiR-101 promotes pain hypersensitivity in rats with chronic constriction injury via the MKP-1 mediated MAPK pathway. J Cell Mol Med.

[CR29] Sayo A (2019). GPR34 in spinal microglia exacerbates neuropathic pain in mice. J Neuroinflamm.

[CR30] Skaper SD, Facci L, Zusso M, Giusti P (2017). Neuroinflammation, mast cells, and glia: dangerous liaisons. Neuroscientist.

[CR31] Torrance N, Smith BH, Bennett MI, Lee AJ (2006). The epidemiology of chronic pain of predominantly neuropathic origin. Results from a general population survey. J Pain.

[CR32] Tunnicliffe RB (2019). Molecular mechanism of SR protein kinase 1 inhibition by the herpes virus protein ICP27. mBio.

[CR33] van Hecke O, Austin SK, Khan RA, Smith BH, Torrance N (2014). Neuropathic pain in the general population: a systematic review of epidemiological studies. Pain.

[CR34] Wong SSC, Lee UM, Wang XM, Chung SK, Cheung CW (2019). Role of DLC2 and RhoA/ROCK pathway in formalin induced inflammatory pain in mice. Neurosci Lett.

[CR35] Wu J, Wang C, Ding H (2019). LncRNA MALAT1 promotes neuropathic pain progression through the miR1545p/AQP9 axis in CCI rat models. Mol Med Rep.

[CR36] Xia LX, Ke C, Lu JM (2018). NEAT1 contributes to neuropathic pain development through targeting miR-381/HMGB1 axis in CCI rat models. J Cell Physiol.

[CR37] Xie T, Zhang J, Kang Z, Liu F, Lin Z (2019). miR-101 down-regulates mTOR expression and attenuates neuropathic pain in chronic constriction injury rat models. Neurosci Res.

[CR38] Yan XT (2018). XIST accelerates neuropathic pain progression through regulation of miR-150 and ZEB1 in CCI rat models. J Cell Physiol.

[CR39] Yasui M (2014). A chronic fatigue syndrome model demonstrates mechanical allodynia and muscular hyperalgesia via spinal microglial activation. Glia.

[CR40] Yousuf MS (2020). Endoplasmic reticulum stress in the dorsal root ganglia regulates large-conductance potassium channels and contributes to pain in a model of multiple sclerosis. FASEB J.

[CR41] Zhang Y (2014). Peripheral pain is enhanced by insulin-like growth factor 1 through a G protein-mediated stimulation of T-type calcium channels. Sci Signal.

[CR42] Zhang D, Mou JY, Wang F, Liu J, Hu X (2019). CRNDE enhances neuropathic pain via modulating miR-136/IL6R axis in CCI rat models. J Cell Physiol.

[CR43] Zhang W, Liu S, Liu K, Liu Y (2019). Long non-coding RNA deleted in lymphocytic leukaemia 1 promotes hepatocellular carcinoma progression by sponging miR-133a to regulate IGF-1R expression. J Cell Mol Med.

[CR44] Zhang T, Yang J, Gong F, Li L, Li A (2020). Long non-coding RNA CASC9 promotes the progression of retinoblastoma via interacting with miR-145-5p. Cell Cycle (Georgetown, Tex).

[CR45] Zheng D (2016). MicroRNA-511 binds to FKBP5 mRNA, which encodes a chaperone protein, and regulates neuronal differentiation. J Biol Chem.

