# Supplementary figures and images for: Downregulation of long noncoding RNA DLEU1 attenuates hypersensitivity in chronic constriction injury-induced neuropathic pain in rats by targeting miR-133a-3p/SRPK1 axis
Source: Mol Med. 2020 Nov 10;26:104. doi: 10.1186/s10020-020-00235-6 (PMC7653812; doi:10.1186/s10020-020-00235-6)

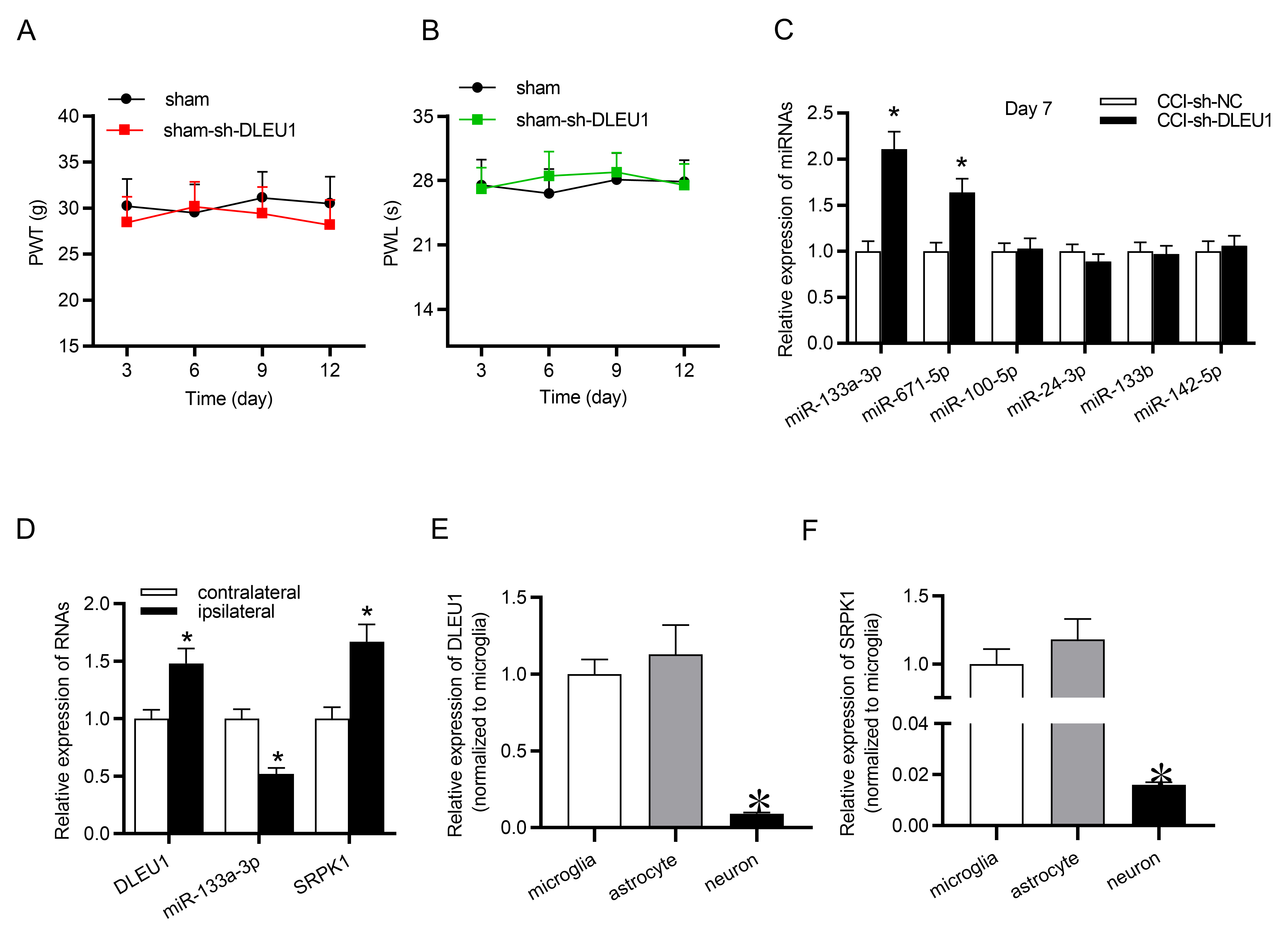

Supplement: Supplementary file 1 — Additional file 1: Fig. S1. (A-B) PWT and PWL were evaluated in DLEU1 silenced sham rats (compared to sham rats without any other treatments) on Day 3, 6, 9, 12 post delivery of LV-sh-DLEU1. N=8 for each group. (C) Relative expression of 6 candidate miRNAs in spinal cord tissues of rats at postoperative day 7 of CCI was examined by RT-qPCR analysis. N=8 for each group. (D) Relative expression of DLEU1, miR-133a-3p and SRPK1 in spinal cord dorsal horn contralateral to the CCI surgery (compared to ipsilateral segments) was examined by RT-qPCR analysis. N=8 for each group. (E-F) Relative expression of DLEU1 and SRPK1 in astrocytes and neuronal cells (compared to microglia) isolated from CCI rats was detected by RT-qPCR analysis. *p < 0.05. [file 10020_2020_235_MOESM1_ESM.tif]
